# Analysis of Pain and Interference Patterns With Brief Pain Inventory in Patients With Bone Metastases: A Confirmatory Study

**DOI:** 10.4021/wjon322w

**Published:** 2011-06-08

**Authors:** Liang Zeng, Edward Chow, Liying Zhang, Shaelyn Culleton, Lori Holden, Florencia Jon, Luluel Khan, Cassandra Uy, May Tsao, Elizabeth Barnes, Cyril Danjoux, Arjun Sahgal

**Affiliations:** a Rapid Response Radiotherapy Program, Department of Radiation Oncology, Odette Cancer Center, Sunnybrook Health Sciences Center, University of Toronto, Canada

**Keywords:** Bone metastases, Pain evaluation, Functional interference, Brief Pain Inventory

## Abstract

**Background:**

This study investigates the validity of the psychometric properties of the Brief Pain Inventory (BPI) in patients with bone metastases and determines if patients with lower body pain exhibit higher levels of activity interference than those with upper body pain.

**Methods:**

Three hundred and eighty-six patients treated, between May 2003 and June 2007, for painful bone metastases were included in this analysis, 336 patients with complete data were included in further analyses. Cronbach’s Alpha, confirmatory factor analysis (CFA), and discriminant validity tests were performed to analyze the psychometric properties of the BPI. One-way analysis of variance was used to compare mean scores of BPI subscales (pain, activity, and affect) in patients with upper or lower bone metastases.

**Results:**

Internal consistency of two- and three-factor BPI analysis was high. In both cases, consistency was further improved when the sleep item was removed. CFA confirmed these results and showed that three-factor analysis was recommended. Patients with lower body metastases reporting moderate to severe pain exhibited greater levels of functional interference. A single fraction radiotherapy dose of 8 Gy was as effective as multi-fraction therapy where the predominant fractionation was 20 Gy in 5 fractions.

**Conclusions:**

Our data confirms the psychometric validation of the BPI and the recommendations to use three-factor analysis in patients with bone metastases. Patients exhibiting lower extremity pain should receive prompt pain interventions and functional aid.

## Introduction

Approximately 50 - 75% of patients with advanced cancer will develop bone metastases and the pain that results is often the first sign of disseminated disease [1]. Common cancers that spread to the bone include that of the breast, prostate and lung [2]. Although numerous skeletal related events such as hypercalcemia, pathological fractures and spinal cord compression [3] can occur as a result of bone metastases, pain is the most prevalent symptom which reduces quality of life and increases analgesic use.

Treatment for bone metastases includes orthopedic interventions (such as minimally invasive procedures and surgery), radionuclides, systemic therapies, conventional radiotherapy [4], stereotactic body radiotherapy [5], and analgesic therapy [6]. Conventional radiotherapy is the most common therapeutic modality to palliate pain associated with bone metastases. In the past, studies investigating conventional radiotherapy have generally used pain and analgesic consumption as the main endpoint [4, 6-9] and rarely examined functional interference. Physical and emotional functional outcomes have been recognized as important endpoints to be considered in the evaluation of a therapeutic modality and were highlighted by the Initiative on Methods, Measurement, and Pain Assessment in Clinical Trials (IMMPACT) recommendations [10]. Previous literature has also failed to determine if location of the site of bone metastases could impact both pain and functional interference. Recent research suggests that patients with lower body painful metastatic disease exhibit higher levels of functional interference [11] and this observation requires confirmation.

The purpose of this study is to validate the psychometric properties of the Brief Pain Inventory (BPI) for patients with bone metastases and determine if patients with lower skeletal pain exhibit higher levels of functional interference than those with upper skeletal pain.

## Methods

### Demographics

The Rapid Response Radiotherapy Program (RRRP) at the Odette Cancer Center, Sunnybrook Health Sciences Center, Toronto, Ontario, Canada is a dedicated palliative radiotherapy clinic designed to provide quick access to palliative radiotherapy. Patients seen at the RRRP between May 2003 and June 2007 were screened for eligibility into a prospective research ethics board-approved study designed to evaluate both pain and functional interference in patients with painful bone metastases treated with conventional palliative radiotherapy using the BPI assessment tool.

### BPI

The BPI is a multidimensional self-assessment tool commonly used to evaluate cancer pain [12, 13]. It has been validated internationally [14-18] and, in previous analyses, has demonstrated construct validity in two IMMPACT recommendations: pain interference and pain intensity. Although it has often been used to screen symptoms in cancer patients, the psychometric validity of the tool has not been confirmed in patients with bone metastases until recently [11]. The BPI lists three questions regarding pain intensity and seven regarding pain interference, anchored between a scale of zero (no pain/interference) to ten (maximum pain/interference). Functional interference scales assess how pain has affected general activity, mood, walking ability, normal work, relations with others, sleep, and enjoyment of life.

### Statistical analysis

Demographic results were expressed as mean, standard deviation (SD), median and inter-quartiles for continuous variables, and proportion for categorical variables. Ideally, BPI assessments would be completed for all patients; however, there were some patients with missing values in one or more interference items. Patient characteristics between those who completed the BPI instrument in its entirety (study patients) versus those with one or more missing items (excluded patients) were compared using Wilcoxon rank-sum test (for continuous variables) or Fisher exact test (for categorical variables).

### Item analysis and internal consistency

The three pain items (worst pain, average pain and current pain) and seven BPI functional interference items were categorized as pain and interference respectively (two factor analysis). Interference was further separated into subscales of activity and affect (three factor analysis). The activity subscale includes the BPI items regarding interference with general activity, walking ability, work and sleep, while the affect subscale includes interference with mood, enjoyment of life and relations with others. Within each subscale, item-item correlations were examined to identify redundant questions. Internal consistency was examined in two (pain and interference) and three subscales (pain, activity, and affect). Standardized Cronbach’s alpha was applied to estimate internal consistency within each subscale [19]. The standardized alpha coefficient provides information about how each BPI item reflects the reliability of the subscale with standardized items. Further changes in Cronbach’s alpha were determined within each subscale after removing individual items. If the standardized alpha decreases after removing a BPI item from the construct, then this item is strongly correlated with other items in the subscale. On the other hand, if the standardized alpha increases after removing a BPI item from the construct, then removing this item from the subscale makes the construct more reliable.

### Confirmatory factor analysis (CFA)

Confirmatory factor analysis was used to examine the scale structure of the BPI as a single construct (one factor), two-factor and three-factor models [20]. One-factor model is the null model of a 10-item single factor model, the two-factor model includes pain and interference subscales and the three-factor model includes the pain, activity and affect subscales. Covariance terms for the two-factor and three-factor modeling were included using the following steps: 1) with a default structure; 2) with covariance of error terms between average pain and current pain, between mood and relations, between mood and enjoyment of life, and between relations and enjoyment of life; 3) using the previous covariance error term structure with removal of the sleep item [11]. Models were compared using various model fit statistics [21] including: 1) adjusted goodness of fit index, which examines the ability of the model to explain the variance in the sample covariance matrix (perfect fit = 1), analogous to corrected R-square; 2) Chi-square statistic (i.e., likelihood ratio Chi-square), which represents the value of the statistical criterion minimized in maximum likelihood estimation (smaller value = better fit); 3) root mean square error of approximation (RMSEA) with 90% upper level of confidence intervals - RMSEA measures the lack of fit of the model to the population covariance matrix (RMSEA ≥ 0.10 suggests poor fit); 4) Bentler’s comparative fit index (CFI) and non-normed fit index (NNFI), which measure the improvement in the overall fit and model complexity (above 0.9 suggest acceptable model fit).

Standardized factor loadings, associated statistics (i.e., R-Squared and t-statistic), and composite reliability were provided for the best two-factor and three-factor models. Composite reliability is a measure of the overall reliability of a collection of heterogeneous but similar items. It is defined as Sum(A)^2^/[Sum(A)^2^ + Sum(B)], where sum(A) indicates sum of standardized factor loading and sum(B) indicates sum of measurement error (i.e., 1 - factor loading). The minimum acceptance level of composite reliability is 0.70, and the minimum critical t value is 3.29 for P = 0.001.

Discriminant validity tests (Chi-square difference test, confidence interval test and variance extracted test) were carried out to further evaluate highly correlated factors within the three-factor model. The confidence interval, including the value of 1.0, indicates significance. The variance extracted estimates the amount of variance that is explained by an underlying factor in relation to the amount of variance due to measurement error. Fornell and Larcker suggested that constructs should exhibit estimates of 0.50 or larger [22]. It should be noted that Hatcher [20] cautions that the variance extracted estimate test is conservative; reliabilities can be acceptable even if variances extracted estimates are less than 0.50.

### Analysis of upper and lower skeletal pain

Patients who received radiotherapy to the lumbar spine, sacrum, or any of pelvic girdle (iliac wing, acetabulum, pubic bone, and ischial tuberosity), femur (head, neck, or shaft), and tibia were defined as having “lower skeletal pain”. Patients who were irradiated to the cervical or thoracic spine, shoulder girdle/upper extremity, ribs, and skull were grouped as those having “upper skeletal” bone pain. Those receiving radiotherapy to the thoracolumbar spine were excluded from this analysis as a potential confounder given that disease involving both thoracic and lumbar vertebrae may not be reliably categorized as upper vs. lower pain. One-way analysis of variance (ANOVA) was used to compare mean scores of BPI subscales (pain, activity, and affect) in patients with upper vs. lower bone metastases. Pain subgroups were further stratified according to the following severity index: mild (≤ 4), moderate (5 - 6), and severe (≥ 7). Test of heterogeneity of effect on mean activity scores between pain subgroups and upper/lower skeletal groups (interaction term) were conducted by general linear model.

All analyses were performed using Statistical Analysis System (SAS version 9.2 for Windows) software. Confirmatory factor modeling was carried out using SAS covariance analysis of linear structural equations (PROC CALIS) procedure. A two-sided P-value of less than 0.05 was considered as statistically significant.

## Results

### Patient characteristics

A total of 386 patients had some form of baseline and demographic information in our database and 336 (87.3%) had complete information. The most commonly missed item on the BPI was normal work (29 patients) and least commonly missed was worst pain (1 patient). Only one patient was excluded for reporting all pain scores as zero. A comparison between study and excluded patient characteristics is shown in Table 1. Older patients (P = 0.013), those with lower KPS (P < 0.0001), and males (P = 0.03) were more likely to have missing responses. Pain scores, morphine equivalence, primary cancer and pain sites were not determinants for patient exclusion in the study.

**Table 1:** Baseline Demographic Information on Study Patients and Excluded Patients

	Study Patients (n = 336)	Excluded Patients (n = 49)	P-value
Age (year)			0.0131
N	336	49	
Mean ± SD	65.3 ± 12.0	69.6 ± 12.7	
Inter-quartiles	57 - 74	61 - 79	
Median (range)	67 (30 - 91)	71 (33 - 90)	
KPS			< 0.0001
N	323	49	
Mean ± SD	71.3 ± 13.4	63.3 ± 11.1	
Inter-quartiles	60 - 80	60 - 70	
Median (range)	70 (30 - 90)	60 (30 - 90)	
Worst pain			0.9859
N	336	48	
Mean ± SD	7.38 ± 2.34	7.48 ± 2.05	
Inter-quartiles	6 - 10	6 - 9	
Median (range)	8.0 (2 - 10)	8.0 (3 - 10)	
Average pain			0.7995
N	336	42	
Mean ± SD	4.98 ± 2.30	5.10 ± 2.15	
Inter-quartiles	3 - 7	3 - 7	
Median (range)	5.0 (0 - 10)	5.0 (1 - 10)	
Current pain			0.3684
N	336	46	
Mean ± SD	3.78 ± 2.78	3.33 ± 2.46	
Inter-quartiles	2 - 6	2 - 5	
Median (range)	3.0 (0 - 10)	3.0 (0 - 10)	
Total daily morphine equivalent (mg)			0.1555
N	333	49	
Mean ± SD	95.5 ± 216.5	137.8 ± 232.5	
Inter-quartiles	0 - 100	0 - 150	
Median (range)	20 (0 - 2600)	40 (0 - 1056)	
Gender			0.0303
Male	192 (57.1%)	36 (73.5%)	
Female	144 (42.9%)	13 (26.5%)	
Dose/Fraction			0.8437
8 Gy/1	209 (62.2%)	31 (63.3%)	
20 Gy/5	111 (33.0%)	15 (30.4%)	
30 Gy/10	7 (2.1%)	1 (2.0%)	
Others	9 (2.7%)	2 (4.1%)	
Primary cancer site			0.3132
Breast	90 (26.8%)	9 (18.4%)	
Prostate	82 (24.4%)	16 (32.6%)	
Lung	85 (25.3%)	9 (18.4%)	
Bladder	13 (3.9%)	1 (2.0%)	
Pancreas/Gastric	10 (3.0%)	3 (6.1%)	
Others	56 (16.7%)	11 (22.4%)	
Pain site			0.6352
Lower limb	152 (45.2%)	20 (40.8%)	
Spine	112 (33.3%)	17 (34.7%)	
Upper limb	42 (12.5%)	6 (12.2%)	
Other - ribs	22 (6.6%)	3 (6.1%)	
Other - chest wall	8 (2.4%)	3 (6.1%)	

The median age of the 336 study patients was 67 years (range 30 - 91) and most prevalent primary cancers were breast (27%), prostate (24%) and lung (25%) (Table 1). The median worst pain was 8.0 (range 2 - 10), average pain was 5.0 (range 0 - 10) and patients were receiving a median of 20 mg/day of oral morphine equivalency.

### Item analysis and internal consistency of subscales

Among 336 study patients, worst, average and current pain had median values of 8.0, 5.0 and 3.0, respectively. With respect to interference, pain interfered most with general activity (median 7.0; range 0 - 10), walking ability (median 7.0; range 0 - 10), normal work (median 8.0; range 0 - 10) and enjoyment of life (median 7.0; range 0 - 10).

The internal consistency of both two and three subscales was high (Table 2). The standardized Cronbach’s alpha was close to 1.0 for two subscales (pain and interference): 0.76 for pain subscale and 0.87 for interference subscale. In the three subscales (pain, activity, and affect), alpha was 0.76 for pain, 0.81 for activity, and 0.77 for affect. When sleep was removed from the activity and interference subscales, Cronbach’s alpha increased further to 0.83 and 0.87, respectively. The fact that sleep demonstrated low correlation with other BPI items indicates that the removal of sleep could make both models more reliable.

**Table 2:** Summary of BPI Scores by Item and Internal Consistency Among Subscales in Study Patients (N = 336)

BPI Items	Item Statistics		Standardized Cronbach’s alpha			
			Two Subscales		Three Subscales	
	Mean	SD	Correlation With Total	Alpha With Item Deleted	Correlation With Total	Alpha With Item Deleted
			Pain Subscale (alpha = 0.76)		Pain Subscale (alpha = 0.76)	
Worst pain	7.4	2.3	0.57	0.71	0.57	0.71
Average pain	5.0	2.3	0.68	0.59	0.68	0.59
Current pain	3.8	2.8	0.54	0.75	0.54	0.75
			Interference Subscale (alpha = 0.87)		Activity Subscale (alpha = 0.81)	
General activity	6.5	3.2	0.77	0.83	0.74	0.70
Walking ability	5.8	3.6	0.59	0.86	0.60	0.78
Normal work	6.8	3.5	0.71	0.84	0.71	0.72
Sleep	4.8	3.4	0.52	0.87↑	0.47	0.83↑
					Affect Subscale (alpha = 0.77)	
Mood	5.1	3.4	0.66	0.85	0.65	0.63
Enjoyment of life	6.5	3.3	0.73	0.84	0.56	0.73
Relations	3.4	3.5	0.53	0.87	0.59	0.70

↑ Removing the sleep item caused alpha to increase in both two and three subscale analysis, meaning it correlated poorly with the other BPI items.

### Confirmatory factor analysis

Both the null, 10-item single factor and the established two-factor default model demonstrated poor fit, the latter being slightly better than the first. By removing the sleep item and allowing specific error terms to co-vary from the analysis, the two-factor and three-factor models were markedly improved (Table 3). Adjusted goodness of fit index was 0.98 (near to perfect fit of 1), RMSEA was 0.04 (< 0.10), CFI was 0.99 (> 0.90), and NNFI was 0.99 (> 0.90) for both two-factor and three-factor models. Chi-square analysis using the two-factor model prior to sleep removal was 46.1. This value improved to 29.7 when the item was removed. Similarly, in the three-factor analysis, Chi-square improved from 46.0 to 28.2 after the sleep item was removed. When considering factor loadings and associated statistics for the best two-factor and three-factor models with sleep removed, all factor loadings were highly significant with t-value between 9.6 and 25.9 (minimum critical t value of 3.29 for P = 0.001) (Table 4). Indicator reliability of individual items (R-squared) ranged from 0.22 (relations) to 0.78 (general activity). There was high internal consistency with regards to composite reliability of the factors in both models, ranging from 0.70 to 0.83 (minimum acceptable level = 0.70). The correlation between pain and interference subscale in the two-factor model was 0.77. In the three-factor model, the correlation between pain and activity was 0.75; between pain and affect was 0.76; and between activity and affect was 0.92. The higher correlation between activity and affect possibly demonstrates the same latent variable.

**Table 3:** Comparison of Model Fit of Different Structural Models

Model	Modification	Goodness of Fit Index (Adjusted)	Chi-Square (df)	RMSEA (Upper CL)	Comparative Fit Index	Non-Normed Fit Index
1 factor: 10 items	None (no structure)	0.88	210.7 (34)	0.12 (0.14)	0.88	0.84
2 factors: Pain and Interference	None (default structure)	0.93	126.3 (32)	0.09 (0.11)	0.94	0.91
2 factors: Pain and Interference	Covary error terms of average pain–current pain, mood–relations, mood–enjoyment, relations–enjoyment	0.97	46.1 (28)	0.04 (0.07)	0.99	0.98
2 factors: Pain and Interference	Drop Sleep; Covary error terms of average pain–current pain, mood–relations, mood–enjoyment, relations–enjoyment	0.98	29.7 (20)	0.04 (0.07)	0.99	0.99
3 factors: Pain, Activity and Affect	None (load sleep on activity)	0.94	111.3 (29)	0.09 (0.11)	0.95	0.91
3 factors: Pain, Activity and Affect	Covary error terms of average pain–current pain, mood–relations	0.97	46.0 (27)	0.05 (0.07)	0.99	0.98
3 factors: Pain, Activity and Affect	Drop Sleep; Covary error terms of average pain–current pain, mood–relations	0.98	28.2 (19)	0.04 (0.07)	0.99	0.99
Acceptable model fit		Perfect fit = 1	Smaller value	RMSEA < 0.10	> 0.90	> 0.90

df = degrees of freedom; CL = confidence limit

**Table 4:** CFA With Sleep Interference Excluded Demonstrating Comparable Factor Loadings and Reliability of Both Two- and Three-Factor Models

BPI Items	Two Subscales			Three Subscales		
	Factor Loading	R-Squared	t-statistic	Factor Loading	R-Squared	t-statistic
	Composite Reliability Pain Factor 0.72			Composite Reliability Pain Factor 0.70		
Worst pain	0.73	0.72	21.1	0.72	0.73	21.0
Average pain	0.76	0.49	16.5	0.74	0.48	16.2
Current pain	0.53	0.24	9.8	0.50	0.24	9.7
	Composite Reliability Interference Factor 0.83			Composite Reliability Activity Factor 0.77		
General activity	0.82	0.77	27.6	0.83	0.78	25.9
Walking ability	0.63	0.47	16.6	0.63	0.47	16.3
Normal work	0.71	0.61	21.3	0.72	0.62	20.5
				Composite Reliability Affect Factor 0.70		
Mood	0.62	0.37	14.0	0.66	0.42	16.9
Enjoyment of life	0.75	0.63	22.6	0.77	0.72	25.3
Relations	0.50	0.22	9.6	0.56	0.27	11.7
	Correlation Pain-Interference: 0.77			Correlation Pain-Activity: 0.75		
				Correlation Pain-Affect: 0.76		
				Correlation Activity-Affect: 0.92		

Discriminant validity was used to analyze activity and affect and overall showed varying results. Chi-square difference test revealed a value of 18.5 (df = 1, P < 0.001), indicative of significance between mixed and separated support. The confidence intervals demonstrated significance (correlation 0.92, 95% confidence interval 0.86 - 0.97). The square of the correlation between activity and affect was 0.88, which was greater than both variance extracted estimates for affect (0.54) and activity (0.78). If the variance extracted estimates were higher than the square of the correlation, it would have shown successful discriminant validity. However, the variance extracted test failed to confirm the validity. Ultimately, the combination of different validity analyses confirmed the validity of the construct.

From above results and the reasonably high levels of internal consistency, composite reliability and discriminant validity can be demonstrated in both two-factor and three-factor models. Overall, the three-factor model with removal of sleeping item was preferred because of fewer covariance terms for fitting the model, and its clinical importance in differentiating patients with different sources of pain.

### Pattern of pain, activity, and affect interference in lower vs. upper skeletal pain

Among the 336 patients with completed data, 199 had lower body and 130 had upper body skeletal metastases treated with palliative radiotherapy; seven patients were excluded as their radiation field covered the thoracolumbar spine. Mean pain scores were not significantly different between both patient groups (Table 5) (P = 0.346); however, patients with lower skeletal metastases as compared to upper skeletal metastases were more likely to exhibit activity interference (mean of 6.9 vs. 5.7, P = 0.0005). With respect to affect, there was no significant difference between these two groups (P = 0.817). When analyzing mean activity scores in upper and lower skeletal patient groups stratified by pain severity index groups (mild, moderate and severe), there was a significantly higher mean activity interference among patients in the lower skeletal group with moderate or severe pain, but not among patients with mild pain (Table 6). Patients in the upper skeletal group showed no significant correlation between activity scores and pain severity. Radiotherapy dosage had no correlation with pain relief as those receiving a single fraction of 8 Gy exhibited the same outcomes as those who received multiple fractions (predominantly 20 Gy in 5 fractions).

**Table 5:** Summary of Subscale Mean Scores in Patients With Upper vs. Lower Skeletal Pain

Subscales	Upper Skeletal Pain (n = 130)		Lower Skeletal Pain (n = 199)		p-value	Mean Difference (95% CI)
	Mean	SD	Mean	SD		
Mean Pain	5.25	1.91	5.47	2.08	0.3461	0.21 (-0.23 - 0.66)
Mean Activity	5.70	2.82	6.85	2.94	0.0005	1.14 (0.50 - 1.79)
Mean Affect	5.05	2.74	4.97	2.83	0.8172	0.07 (-0.55 - 0.69)

**Table 6:** Mean Activity Scores in Upper and Lower Skeletal Patient Groups Stratified by Pain Subgroups

Pain subgroups	Upper Skeletal Pain (n = 130)		Lower Skeletal Pain (n = 199)		p-value	Mean Difference (95% CI)
	n	Mean (SD)	n	Mean (SD)		
Mild (mean Pain ≤ 4)	35	4.03 (2.69)	51	4.14 (3.20)	0.8617	0.12 (-1.20 - 1.43)
Moderate (mean Pain 4 - 7)	69	6.03 (2.74)	101	7.37 (2.28)	0.0007	1.34 (0.57 - 2.10)
Severe (mean Pain ≥ 7)	26	7.08 (2.14)	47	8.65 (1.66)	0.0008	1.58 (0.68 - 2.47)

## Discussion

Research regarding functional interference resulting from pain in advanced cancer patients is lacking. The BPI is a common tool used to assess functional interference; however, the psychometric properties have not been validated in patients with bone metastases until recently. Wu et al. conducted an analysis of 258 patients receiving radiotherapy for painful bone metastases and suggested that the three-factor analysis of the BPI with the sleep item removed is preferred and patients with lower body pain will experience higher levels of functional interference than those with upper body pain [11]. In this analysis, we confirm: a) the psychometric validity of the BPI in patients with bone metastases, b) that sleep does not correlate well with the other items and c) that patients with lower body pain exhibit higher levels of functional interference than those with upper body pain.

Our analysis of the psychometric properties of the BPI is compared to that by Wu et al. in Table 7, and confirms their findings. In both studies, greater consistency was demonstrated in the BPI when the sleep item was removed. Furthermore, in both analyses of model fit, the three-factor model was superior over the null and two-factor model with removal of the sleep item. In both studies, correlation between activity and affect was very high, possibly representative of the same latent variables (Table 7). Overall, three-factor analysis contains fewer covariance terms and satisfies an additional IMMPACT recommendation and therefore should be the preferred form of analysis in this group of patients.

**Table 7:** Comparison of Wu et al. and Our Results (Three-Factor Analyses)

Three-Factor Test	Wu et al. [11]	Our Results
Cronbach’s Alpha		
Initial Activity Alpha	0.79	0.81
Activity Alpha Removing Sleep	0.89	0.83
Result	Increase	Increase
Model Fit		
Chi-square with sleep	152.6	46.0
Chi-square without sleep	37.9	28.2
Result	Decrease	Decrease
Model CFA		
Correlation pain-activity	0.52	0.75
Correlation pain-affect	0.55	0.76
Correlation activity-affect	0.83	0.92

The improvement in the model with removal of the sleep item makes sense as the relationship between sleep and pain is very complex in nature. Literature has identified predisposing and perpetuating factors that may lead to insomnia [23], and emotional, socio-cultural, environmental, and religious factors all may influence sleep quality [24]. The fact that sleep does not correlate with other items of the BPI may be surprising in this highly symptomatic population, but an explanation could be that the origin of insomnia varies greatly on a patient-to-patient basis. Furthermore, the use of sleep medications was not taken into account in follow-up assessments or in the oral morphine equivalent calculation. Varying relationships have been previously reported between sleep and the other items of the BPI [11, 13, 25]. For example, Cleeland et al. proposed that sleep interference would be related closest to the activity dimension, Klepstad et al. [18] suggested that sleep be part of affect, and Saxena et al. recommended the sleep item should be removed completely [25]. Our analysis confirmed findings by Wu et al., indicating the inconsistency with sleep’s correlation among other BPI items and suggesting its exclusion in both the two- and three-factor analysis.

In our analysis of upper versus lower skeletal pain, the latter group suffered more from activity interference, however, only when their pain was categorized as being moderate or severe. In the analysis by Wu et al., only those with mild and moderate pain had significant differences in interference. This discrepancy requires more investigation but suggests that clinicians need to be more aware of the functional impact of symptomatic bone metastases located in the lower skeleton.

A further point of research would be to test correlation with other musculoskeletal functional scales, such as the Western Ontario and McMaster University Osteoarthritis Index (WOMAC) [26] and the Musculoskeletal Function Assessment (MFA) [27] questionnaire. One particular limitation in the present study exists with regards to the patient population. The most commonly missed item on the BPI was normal work where 29 patients recorded no score. As shown in Table 1, elderly male patients with decreased performance status were more likely to skip this item. These patients may view themselves as no longer being capable of doing normal work and, therefore, feel they are not required to answer this question. As a result, elderly male patients with decreased performance statuses may not have been represented sufficiently in our population.

In summary, we have obtained similar results to Wu et al. and agree with their findings. The BPI is a valid measurement tool for patients with bone metastases and should be analyzed using three factors as it satisfies three of four IMMPACT recommendations. Sleep interference does not correlate well with activity or affect subgroups and its removal from analysis improves correlation between other BPI items. The sleep item should be further investigated to examine its relationship to bone metastases specific pain. Patients with moderate to severe lower skeletal pain exhibit higher levels of activity interference than those with upper body skeletal pain and therefore should be given prompt treatment and additional assistance due to their increased functional impairment. Although single dosage radiotherapy being as effective as multi-fraction treatment is not new, it is a point currently being emphasized by the American Society for Therapeutic Radiology and Oncology (ASTRO).
